# Comparing the Respiratory Compensation Point With Muscle Oxygen Saturation in Locomotor and Non-locomotor Muscles Using Wearable NIRS Spectroscopy During Whole-Body Exercise

**DOI:** 10.3389/fphys.2022.818733

**Published:** 2022-03-24

**Authors:** Assaf Yogev, Jem Arnold, Dave Clarke, Jordan A. Guenette, Ben C. Sporer, Michael S. Koehle

**Affiliations:** ^1^Environmental Physiology Laboratory, School of Kinesiology, University of British Columbia, Vancouver, BC, Canada; ^2^Department of Biomedical Physiology and Kinesiology and Centre for Cell Biology, Development and Disease, Simon Fraser University, Burnaby, BC, Canada; ^3^Department of Physical Therapy, University of British Columbia, Vancouver, BC, Canada; ^4^Providence Health Care Research Institute, Centre for Heart Lung Innovation, University of British Columbia, Vancouver, BC, Canada; ^5^Vancouver Whitecaps FC, Vancouver, BC, Canada; ^6^Division of Sport Medicine, University of British Columbia, Vancouver, BC, Canada

**Keywords:** muscle oxygenation, near-infrared spectroscopy, respiratory compensation point, breakpoint, wearable, exercise, exercise testing, cardiorespiratory fitness

## Abstract

The relationship between the muscle deoxygenation breakpoint (Deoxy-BP) measured with near-infrared spectroscopy (NIRS), and the respiratory compensation point (RCP) has been well established. This relationship has also been reported using wearable NIRS, however not in locomotor and non-locomotor muscles simultaneously during whole-body cycling exercise. Our aim was to measure muscle oxygen saturation (SmO_2_) using wearable NIRS sensors, and to compare the Deoxy-BPs at each muscle with RCP during a ramp cycling exercise test. Twenty-two trained female and male cyclists completed a ramp exercise test to task intolerance on a cycling ergometer, at a ramp rate of 1 W every 2 s (30 W/min). SmO_2_ was recorded at the subjects’ right vastus lateralis (VL) and right lateral deltoid. SmO_2_ and the Deoxy-BPs were assessed using a piecewise double-linear regression model. Ventilation (V̇_E_) and gas exchange were recorded, and RCP was determined from V̇_E_ and gas exchange using a V-slope method and confirmed by two physiologists. The SmO_2_ profiles of both muscles and gas exchange responses are reported as V̇O_2_, power output (W), and time of occurrence (TO). SmO_2_ profiles at both muscles displayed a near-plateau or breakpoint response near the RCP. No differences were detected between the mean RCP and mean Deoxy-BP from either the locomotor or non-locomotor muscles; however, a high degree of individual variability was observed in the timing and order of occurrence of the specific breakpoints. These findings add insight into the relationships between ventilatory, locomotor, and non-locomotor muscle physiological breakpoints. While identifying a similar relationship between these breakpoints, individual variability was high; hence, caution is advised when using wearable NIRS to estimate RCP in an incremental ramp test.

## Introduction

The close relationship between the respiratory compensation point (RCP) and the muscle deoxygenation breakpoint (Deoxy-BP) measured by near-infrared spectroscopy (NIRS) during incremental ramp cycling test has been well documented in recent years (Murias et al., 2013; Racinais et al., 2014; Fontana et al., 2015; Keir et al., 2015; Iannetta et al., 2017a; Caen et al., 2018). The ability to use changes in muscle oxygenation signals [deoxy(heme), oxy(heme), saturation, etc.] as a proxy for systemic pulmonary response yields far reaching possibilities for improving athletic performance, such as improving pacing strategies, simplifying performance assessment with non-invasive measures, and the potential for better understanding of central and peripheral limitations during exercise. The wider availability of affordable, portable NIRS devices that can be recorded to commercially available sport watches and cycling computers has made it more feasible for athletes, coaches, and teams to use muscle oxygenation as part of their regular training data collection (McManus et al., 2018; Perrey and Ferrari, 2018; Kirby et al., 2021; Rodrigo-Carranza et al., 2021). Wearable NIRS devices are typically based on continuous wave (CW) NIR light technology, which despite having some technical limitations compared to more robust, non-portable NIRS equipment, introduces the significant advantages of cost reduction and portability for field applications (Perrey and Ferrari, 2018).

In 2013, Murias et al. (2013) compared the recently described breakpoint (BP) in the NIRS-derived deoxygenated hemoglobin (∆[HHb]) measurement at the VL with both the gas exchange threshold (GET) and the RCP, in 10 young males and 10 young females during a continuous ramp exercise test on a bicycle ergometer (Murias et al., 2013). They showed strong correlation in V̇O_2_ obtained at the RCP and ∆[HHb]-BP. Other studies demonstrated a strong agreement between RCP and ∆[HHb]-BP both cross-sectionally (Racinais et al., 2014; Fontana et al., 2015; Keir et al., 2015; Inglis et al., 2017; Caen et al., 2018), in a heterogeneous fitness group (Boone et al., 2015), as well as longitudinally (Inglis et al., 2019). Contradicting evidence demonstrated that RCP and ∆[HHb]-BP occur at slightly different %V̇O_2_max (Boone et al., 2015) and to be somewhat dissociated based on power output (PO), when evaluated across time following an exercise training intervention (Caen et al., 2018). This evidence comes mostly from two laboratories. Therefore, more studies are needed to corroborate the presence, or lack thereof, of this correspondence. Several studies observed the relationship between different muscles’ Deoxy-BP during incremental exercise, and short, maximal intensity exercise, and the RCP during cycling and running. Iannetta et al. (2017a) assessed muscle oxygenation heterogeneity across quadricep muscles during a ramp exercise test on a cycling ergometer and compared the oxygenation response of the vastus lateralis (VL), vastus medialis, and rectus femoris muscles with the RCP (Iannetta et al., 2017b). Their findings showed a strong correlation between the pulmonary gas exchange (V̇O_2_) corresponding to the NIRS-derived ∆[HHb]-BP measured across the different muscle sites and the V̇O_2_ at RCP. Similar relationships have been observed between the V̇O_2_ detected at the RCP, and the V̇O_2_ observed at both the VL and respiratory muscle oxygenation breakpoints (Legrand et al., 2007; Contreras-Briceño et al., 2019), during similar cycling ergometer exercise protocols.

Several studies measured inactive forearm muscle oxygenation response to lower limb cycling exercise using ramp exercise tests (Ogata et al., 2004; Tanaka et al., 2006; Shiroishi et al., 2010). Their findings showed accelerated deoxygenation response past the RCP at the inactive forearm. Conversely, muscle oxygenation response in the biceps brachii (BB) and VL were compared during a 30 s maximal running on a treadmill (Manchado-Gobatto et al., 2020). This study found significant differences between these muscle groups during the final 15 s of the effort, with the BB reporting greater dynamic range in percent muscle deoxygenation (76 ± 2 to 31 ± 3) compared with the VL (79 ± 2 to 50 ± 4). These findings lead to further questions about the relationship between the systemic V̇O_2_ response and the non-locomotor muscle deoxygenation response, and whether the latter can be used to accurately estimate the heavy - severe intensity threshold. If it is possible to detect a heavy - severe threshold using either locomotor or non-locomotor muscles, both endurance athletes and their coaches would be able to use it to optimize pacing strategy for specific competitions and environments, without the need to rely on expensive, invasive gas exchange or blood lactate measurements. The use of both lactate and gas exchange for these applications has been well documented, and thresholds such as the RCP, critical power, and maximal lactate steady state have shown strong agreements in the intensity at which they occur (Keir et al., 2015; Iannetta et al., 2018; Inglis et al., 2019). The primary limitations of both methods are a need for controlled environments for both operation and safety, trained personnel to conduct them due to their invasiveness, and expensive running cost. These limitations can be resolved using wearable NIRS, which requires very little experience to operate as data can be recorded using existing sport computers and applications, and can be done both indoors and in real-world training environments and competition at a relatively affordable cost. Therefore, should a muscle deoxygenation breakpoint be detectable with a wearable unit as described previously with other NIRS devices, at an intensity associated with RCP, it may provide a more accessible physiological measure of intensity dependent effects on muscle metabolism, without the need to rely on laboratory equipment or expert interpretation.

It is important to note that differences do exist between wearable CW-NIRS sensors and more technologically advanced, laboratory-based NIRS devices used in previous reports. Meaning results may not be directly transferable between different NIRS devices. These more advanced NIRS equipment may have multiple emitter-receptor optodes, more precise signal resolution, and higher sampling frequency. This improved measurement sensitivity may provide greater accuracy and reliability with absolute measurements, especially when evaluating subjects of heterogeneous tissue composition and fitness characteristics (McManus et al., 2018; Perrey and Ferrari, 2018; Barstow, 2019). To translate findings from stationary NIRS in the lab to portable NIRS in the field, it is important to replicate findings obtained using advanced NIRS technology with those from commercially available portable devices. A recent study compared muscle oxygenation profiles using a wearable NIRS sensor (Humon Hex, Cambridge, MA, United States) with the RCP in elite runners. They reported no differences in V̇O_2_ or PO between the Deoxy-BP measured at the VL muscle and RCP, during an incremental ramp test on a treadmill (Rodrigo-Carranza et al., 2021). However, it is unknown whether this relationship holds in trained cyclists.

The RCP represents a systemic metabolic threshold. If Deoxy-BP is associated with the RCP, then it may not be limited to a local locomotor phenomenon. As such, a breakpoint may be detectable in non-locomotor muscle sites that reflect this systemic shift in metabolic homeostasis. The purpose of this study was 2-fold (1) to compare muscle Deoxy-BP as detected using a commercially available wearable NIRS with the RCP during a ramp exercise test performed on the subjects’ own bicycles, as they would be performing in everyday training. (2) To measure this response at both locomotor and non-locomotor muscle sites. We hypothesized that wearable NIRS sensors would show a breakpoint or plateau-like response near the RCP, both at the locomotor and non-locomotor muscles, and that an association exists between the PO at RCP and muscle deoxygenation during a ramp exercise test on a bicycle ergometer.

## Materials and Methods

### Subjects

Twenty-two trained cyclists (17 males and 5 females, 31 ± 8 yr.; 75 ± 12 kg; and 179 ± 10 cm) volunteered and provided written informed consent to participate in the study. To obtain sufficient power of *β* = 0.8 with *α* = 0.05, an *a priori* sample size calculation was made in G^*^Power software (version 3.1.9.7, Kiel, Germany), using previously reported data from other groups that compared performance parameters for either gas exchange or PO between RCP and muscle oxygenation response (Murias et al., 2013; Boone et al., 2014; Racinais et al., 2014; Keir et al., 2015; Zwaard et al., 2016; Iannetta et al., 2017a, 2018; Caen et al., 2018; Rodrigo-Carranza et al., 2021). This study was approved by the research ethics committee of University of British Columbia and was conducted in accordance with principles established in the Declaration of Helsinki.

### Experimental Design

**Figure 1:**
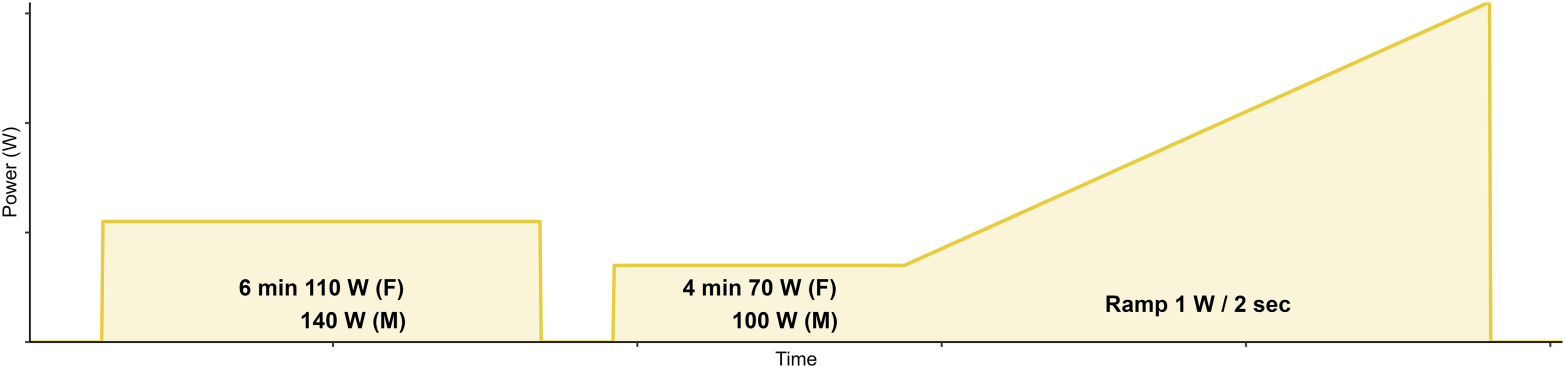
Ramp exercise protocol. An initial baseline warm-up of 6 min at a moderate PO of either 110 W (females) or 140 W (males) was performed. It was then followed by 4 min at 70 W (females) or 100 W (males), before the continuous ramp commenced at 1 W per 2 s. Cessation of exercise was determined by the point at which the participant cadence went down by more than 10 revolutions per minute from their self-selected cadence.

The study required a single visit that included an incremental ramp cycling test from rest to task intolerance completed on an electronically controlled, stationary bicycle trainer (KICKR, Wahoo Fitness Inc., Atlanta, GA, United States) using each participant’s bicycle and riding gear to simulate their regular indoor training environment. The ramp rate increased by 1 W every 2 s (30 W/min; Figure 1), with task intolerance determined as the point at which the participant cadence went down by more than 10 revolutions per minute from their self-selected cadence. Resistance was controlled in ergometer mode using PerfPRO Studio Software^©^ (Hartware Technologies, Rockford, MI, United States) installed on a personal laptop.

Pulmonary oxygen uptake (V̇O_2_) was measured with an open-circuit expired-gas analysis system (TrueOne 2,400; ParvoMedics, Inc., Sandy, UT). V̇O_2_ data were averaged to 15 s and interpolated to 1 Hz for analysis. V̇O_2_peak was considered the highest average 30-s measurement. The RCP was determined at the point of deflection of V̇_E_ relative to V̇CO_2_ and the second deflection of V̇_E_ relative to V̇O_2_ (Davis, 1985; Beaver et al., 1986).

An individual mean response time (MRT) representing the delay between muscular metabolic activity and pulmonary response was determined using a recently described protocol (Murias et al., 2013; Iannetta et al., 2019). Briefly, the subjects performed a baseline warm-up for 6 min at a moderate PO of either 110 W (females) or 140 W (males). Average baseline V̇O_2_ was determined from the final 2 min of the baseline step. The ramp exercise test began with 4 min at 70 W (females) or 100 W (males), before the continuous ramp commenced at 1 W per 2 s. The subject’s V̇O_2_ response during the ramp test was compared to their average baseline V̇O_2_. The difference in the instantaneous PO that elicited the same V̇O_2_ response was used to determine the MRT in watts and in seconds. The MRT was then used to shift PO relative to V̇O_2_ for estimation of the PO that elicited RCP and the V̇O_2_ response at the Deoxy-BP (further described below).

### Near-Infrared Spectroscopy

Three wearable NIRS sensors (Moxy Monitor, Fortiori Design LLC., Hutchinson, United States) were used during the test. The Moxy monitor employs four wavelengths of NIR light (680, 720, 760, and 800 nm), with source detector separation of 12.5 and 25 mm (McManus et al., 2018). The sensors were placed on the following locations: on the vastus lateralis (VL) of the right and left leg, and right lateral deltoid. The right VL (RVL) was used for analysis unless signal quality was poor (*n* = 1), in which case the left side was used. The anatomical location on the VL was 1/3 the distance from the proximal pole of the patella to the greater trochanter. Left and right VL sensors were held in place by the participants’ elastic cycling shorts. The deltoid sensor was positioned on the midline of the lateral deltoid muscle belly inferior to the acromion and maintained in place with a fiber-elastic band. All sensors were covered using a light shield supplied by the manufacturer to minimize interference.

According to the manufacturer, the Moxy sensor does not require calibration. A skinfold measurement with a Harpenden skinfold caliper (Slim Guide Skinfold Caliper) was made on the RVL to ensure all subjects were below 15 mm skinfold thickness, during the initial visit (Perrey and Ferrari, 2018).

### Power Output

Power output was recorded using each participant’s individual onboard power meter. Power meters were zero-offset following manufacturer specifications before each test. The electronic trainer was calibrated immediately after the warm-up of each test to ensure accurate resistance was provided. Peak PO (W_peak_) was defined as the highest 30-s PO recorded during the ramp test.

### Data Collection

All three NIRS sensors and power data were collected using a Garmin Edge 520 cycling computer (Garmin Ltd., Lenexa, United States). Gas exchange and V̇_E_ were collected using Parvo Medics software.

### Data Analysis

Methods used for measuring V̇O_2peak_ and detecting RCP are described above. The Moxy sensor provided measures of estimated total heme concentration ([tHb + Mb] in arbitrary units) and muscle O_2_ saturation (SmO_2_ as a percent). Computation of oxygenated hemoglobin [O_2_Hb + Mb] and deoxygenated hemoglobin [HHb + Mb] can be derived from [tHb + Mb] and SmO_2_ (McManus et al., 2018; Feldmann et al., 2019).

**Figure 2:**
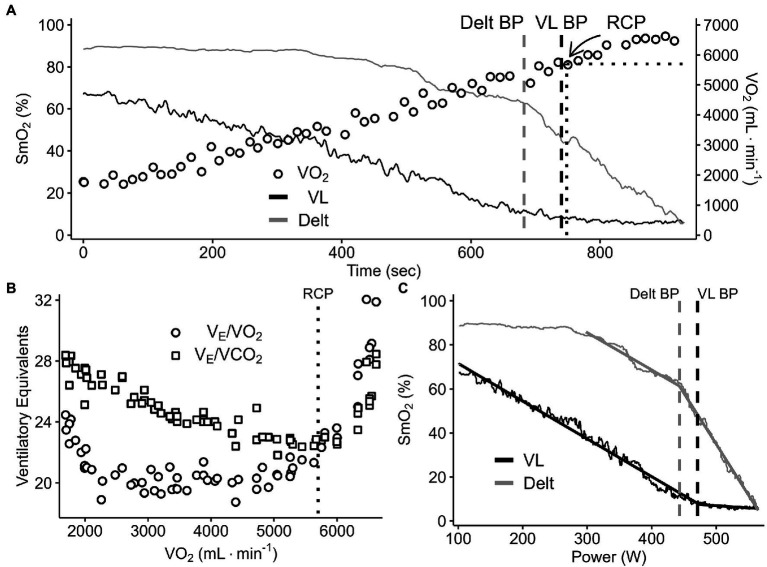
A representative data set of breakpoint detection and comparison between the change in %SmO_2_ (VL and deltoid) and V̇O_2_ (panel **A**), ventilatory equivalents of O_2_ and CO_2_ (panel **B**), and %SmO_2_ VL and deltoid profiles (panel **C**) during a continuous graded exercise test from rest to task intolerance.

The SmO_2_ signal was used as the primary output variable in this study (Crum et al., 2017; McManus et al., 2018; Feldmann et al., 2019). SmO_2_ was measured every 2 s (0.5 Hz) and raw data were smoothed to 5-s moving averages as per manufacturer default settings. The Deoxy-BP was operationally defined as an inflection point in the SmO_2_ profile plotted against time, analogous to the approach using the [HHb + Mb] plateau described by. This gives a time of occurrence (TO) from which the corresponding PO and V̇O_2_ can be determined using the MRT correction detailed above. The breakpoint was located using a piecewise “double-linear” regression model implemented in the training analysis software WKO5 (TrainingPeaks, LLC, Boulder, CO, United States). This method is similar to the V-slope method for detecting RCP from the inflection of V̇_E_ vs. V̇CO_2_ (Davis, 1985; Beaver et al., 1986). Following estimation of both the RCP and Deoxy-BP, each data set was reviewed by two physiologists for confirmation of the RCP and Deoxy-BP detection. Power output, V̇O_2_, and time of occurrence (TO) at the RCP and Deoxy-BP were estimated, accounting for the muscle-pulmonary delay (MRT) as outlined above (Figure 2).

### Statistical Analysis

GraphPad statistical software (GraphPad Software Inc., CA, United States) was used for statistical analysis. To test the hypothesis of non-differences between RCP and the two Deoxy-BP, as well as between the two Deoxy-BP (deltoid and VL), we performed one-way ANOVAs for the three outcome variables (V̇O_2_, PO, and TO) and factors (RCP, Deoxy-BP deltoid, and Deoxy-BP VL). Regression analyses were used to calculate an association between RCP and the two Deoxy-BP, as well as between the two Deoxy-BP (deltoid and VL). Correlation was classified as followed: negligible (0.00–0.30), weak (0.30–0.50), moderate (0.50–0.70), strong (0.70–0.90), or very strong (0.90–1.00; Mukaka, 2012). A post-hoc Tukey Test was employed when significant differences were detected, and a Bland Altman test was used for agreement in the V̇O_2_ between the breakpoints (*p* < 0.05).

## Results

**Figure 3:**
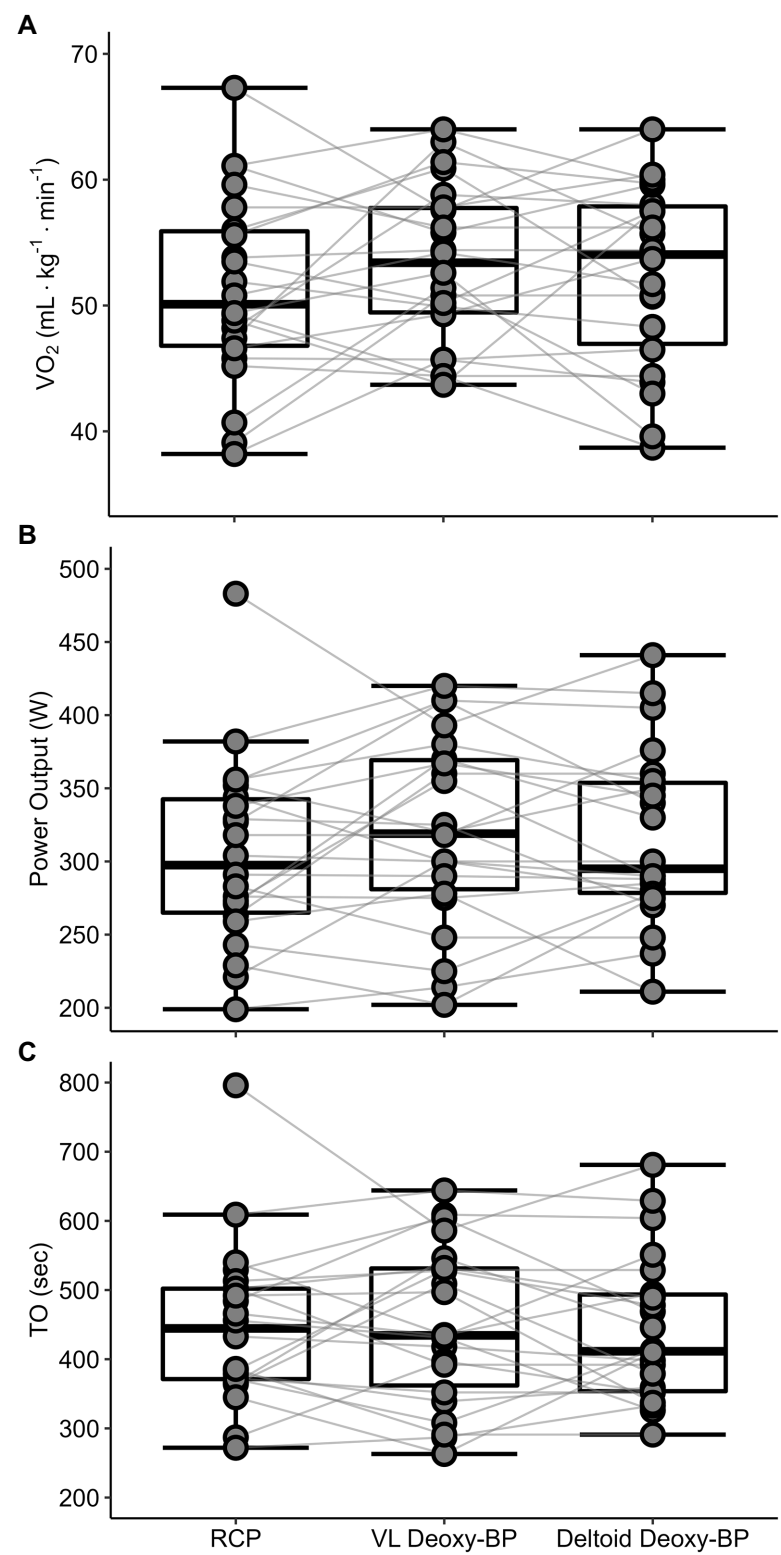
Individual data comparison between RCP, VL, and deltoid Deoxy-BP for V̇O_2_ (ml·kg^−1^·min^−1^; panel **A**), power output (PO in W; panel **B**), and time of occurrence (TO in s; panel **C**).

Means for participant characteristics are presented in Table 1. Means for V̇O_2_, PO, and TO at RCP, and both VL and deltoid Deoxy-BP are presented in Table 2. Mean comparison of RCP with both Deoxy-BP showed no difference in V̇O_2_, PO, or TO (*p* > 0.05). Figures 3A–C presents individual data comparison between the three measurements as a function of V̇O_2_, PO, and TO.

**Table 1:** Characteristics of study participants.

Age (y)	Weight (kg)	Height (cm)	V̇O_2_peak (ml·kg^−1^·min^−1^)	MRT (sec)	W_peak_ (W)	SF (mm)	HR_max_ (bpm)
30.9 ± 8.3	75.0 ± 12.3	178.9 ± 10.1	61.4 ± 11.4	31.1 ± 10.8	406.4 ± 66.1	9.8 ± 4.9	188.9 ± 10.0

Mean data displayed as mean ± standard deviation, for the following variables: age, weigh, height, peak VO_2_ (VO_2__peak_), mean response time (MRT), peak power output (W_peak_), skinfold tissue thickness (SF), and maximum heart rate (HR_max_).

**Table 2:** Mean comparison between respiratory compensation point (RCP), vastus lateralis (VL), and deltoid Deoxy-BP as a function of V̇O2, power output (PO), and time of occurrence (TO).

	RCP	VL	deltoid
V̇O_2_ (ml·kg^−1^·min^−1^)	51.2 ± 7.5	53.3 ± 6.3	52.5 ± 7.3
PO (W)	304 ± 63	321 ± 65	317 ± 60
TO (s)	448 ± 116	451 ± 115	442 ± 106

Mean data displayed as mean ± standard deviation. No significant difference was observed between RCP, VL, and deltoid Deoxy-BP for those variables.

**Figure 4:**
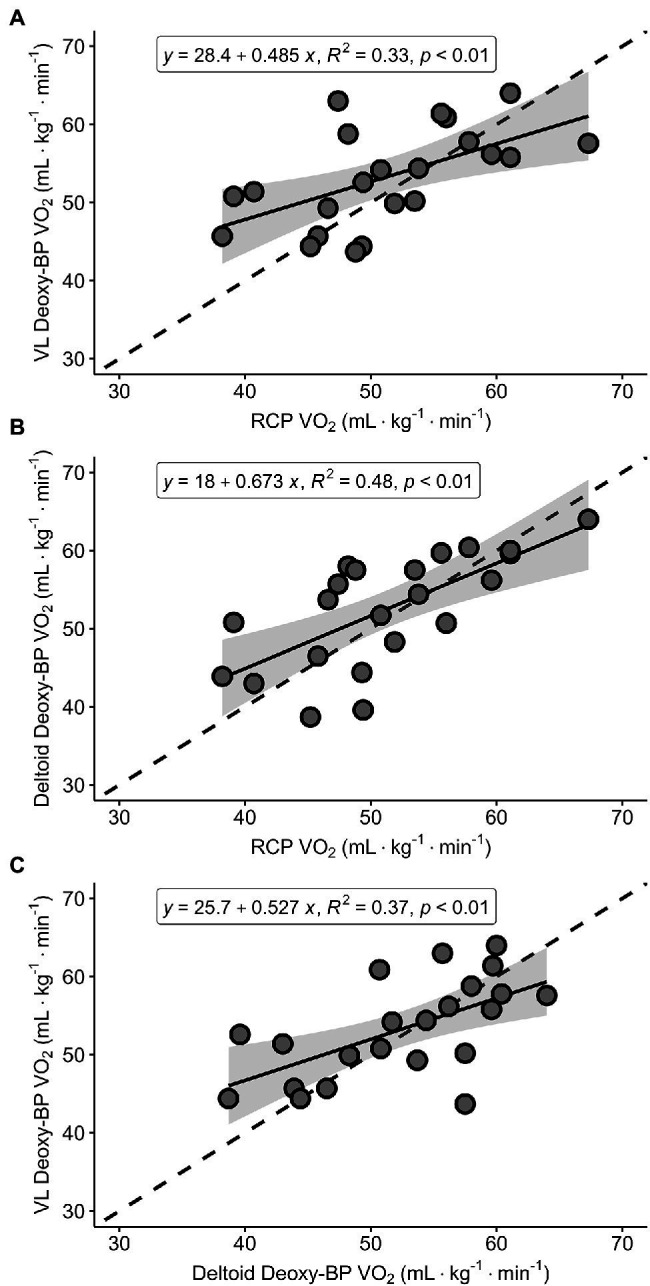
Regression analyses for V̇O_2_ (ml·kg^−1^·min^−1^) between RCP and VL Deoxy-BP (panel **A**), RCP and deltoid Deoxy-BP (panel **B**), and VL Deoxy-BP and deltoid Deoxy-BP (panel **C**).

Figures 4A–C presents Pearson correlations between the V̇O_2_ at RCP and the two Deoxy-BP, as well as between the two Deoxy-BP (VL and deltoid). The relationships across all variables showed positive, moderate relationships (*r* = 0.58–0.69). All correlations were statistically significant (*p* < 0.01).

**Figure 5:**
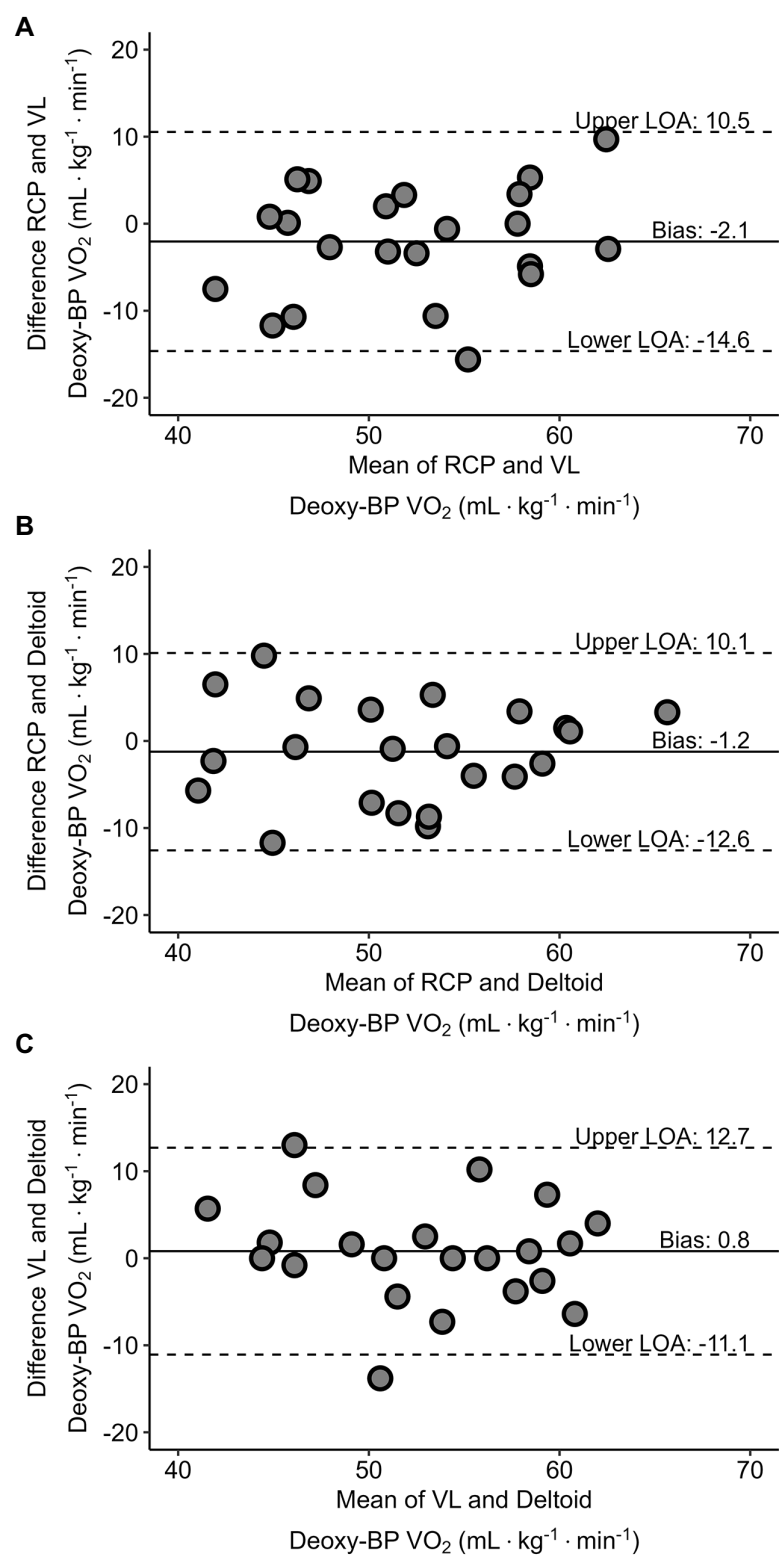
Bland–Altman plots displaying agreement between V̇O_2_ (ml·kg^−1^·min^−1^) corresponding with RCP and VL Deoxy-BP (panel **A**), RCP and deltoid Deoxy-BP (panel **B**), and VL and deltoid Deoxy-BP (panel **C**). The horizontal solid line represents the mean difference between each of the two measurements. The paired horizontal dotted lines represent the 95% limits of agreement for each of the comparisons.

Figures 5A–C presents Bland–Altman plots depicting the agreement between the V̇O_2_ at RCP and both Deoxy-BP. Relative V̇O_2_ (ml·kg^−1^·min^−1^) detected for each of the measurements was used to assess their agreement. The mean average error for RCP and VL Deoxy-BP was −2.1 ml·kg^−1^·min^−1^ (*p* > 0.05; limits of agreement: lower = −14.6 ml·kg^−1^·min^−1^; upper = 10.5 ml·kg^−1^·min^−1^). The mean average error for RCP and deltoid Deoxy-BP was −1.2 ml·kg^−1^·min^−1^ (*p* > 0.05; limits of agreement: lower = −12.6 ml·kg^−1^·min^−1^; upper = 10.1 ml·kg^−1^·min^−1^). Lastly, the mean average error for VL and deltoid Deoxy-BP was 0.8 ml·kg^−1^·min^−1^ (*p* > 0.05; limits of agreement: lower = −11.1 ml·kg^−1^·min^−1^; upper = 12.7 ml·kg^−1^·min^−1^).

## Discussion

The aim of this study was to compare V̇O_2_, PO, and TO corresponding to the RCP, and the breakpoint of muscle oxygenation in the vastus lateralis and deltoid muscles (VL and deltoid Deoxy-BP respectively) measured using wearable NIRS sensors, during a ramp exercise protocol on a bicycle ergometer. From our results, we were unable to detect significant differences across our mean variables which were in support of our main hypothesis. It is important, however, to point out that despite our Bland Altman plots presenting a bias that was not different from zero, they depicted large limits of agreement of > 10 ml·kg^−1^·min^−1^ between the variables. Our findings were in agreement with previous studies that compared the V̇O_2_ and PO at the RCP and at the NIRS-derived Deoxy-BP (Iannetta et al., 2017a,b). Additionally, our results presented that the Deoxy-BP derived from a wearable sensor was not different from the RCP in a group of trained male and female cyclists with heterogeneous fitness. That said, the limits of agreement were poor; hence, caution is advised when using wearable NIRS devices to estimate RCP in an incremental ramp test. This may suggest that the protocol used should be considered when evaluating metabolic thresholds and intensity domains with either gas exchange, blood lactate, or muscle oxygenation. The demarcation of metabolic breakpoints is specific to the assessment protocol and measurement device used.

When all three breakpoints were compared in terms of corresponding V̇O_2_ and PO, no mean differences were observed, and significant moderate to strong correlations between the measures were detected. Previous studies showing no differences used similar analysis; however, a majority of these studies used healthy participants with a lower mean V̇O_2peak_ of approximately 50 ml·kg^−1^·min^−1^ (Iannetta et al., 2017a,b). A study by Boone et al. (2015) had 64 participants with a similar range of heterogeneous fitness as seen in our group, who underwent a similar exercise protocol. Our results showed a similar range at Deoxy-BP at the VL and deltoid when presented in relative V̇O_2_ (ml·kg^−1^·min^−1^). Despite these similarities, our results show large individual variation, which raises concerns about using these indices interchangeably. It is possible that the difference between the findings of Boone, et al., compared with our results is related to the different sensors used for the detection of the Deoxy-BP (laser-based, frequency domain versus CW-NIRS, respectively). Therefore, caution is warranted for practitioners using wearable NIRS sensors when attempting to interpret similar thresholds, using similar exercise protocols.

Although there were no significant differences between the mean V̇O_2_ and PO corresponding to the Deoxy-BP and the RCP, individual data showed that the VL Deoxy-BP and RCP did not occur in a consistent sequence. The difference between the TO for VL Deoxy-BP and RCP ranged from −210 to 180 s, and for deltoid Deoxy-BP and RCP was −155 to 145 (where negative indicates the RCP occurred before the Deoxy-BP, and positive indicates that the Deoxy-BP occurred before the RCP). The TO of the VL Deoxy-BP preceded the RCP TO in 11 of the 22 subjects, and the deltoid Deoxy-BP TO occurred before the RCP TO in 12 subjects, although not necessarily in the same individuals. The inclusion of TO in the analysis was to account for methodological limitations in accounting for MRT (discussed further below) and the differences that might have resulted when shifting either V̇O_2_ or PO to compare the different breakpoints (Murias et al., 2013; Iannetta et al., 2019). In terms of corresponding V̇O_2_, nine subjects had lower V̇O_2_ at their VL Deoxy-BP than at their RCP, and nine subjects (not necessarily the same individuals) had lower V̇O_2_ at their deltoid Deoxy-BP. Such differences have also been shown by Boone et al. between the V̇O_2_ at the RCP and the deoxygenated hemoglobin breakpoint BP deoxy[Hb+Mb] similar to Deoxy-BP used in our study), with the RCP preceding the breakpoint in deoxy[Hb+Mb] during an incremental ramp cycle test (Boone et al., 2015). They suggested two hypotheses for the potential mechanism responsible for this sequence. These hypotheses are focused on (1) muscle metabolism changes during a ramp exercise test stimulating both V̇_E_ and respiratory alkalosis and vasodilation, resulting in increased blood delivery to the working muscles. (2) An increase in less oxidatively efficient type IIa and type IIx muscle fiber recruitment above the RCP to maintain power production in response to the increased resistance (Boone et al., 2015). These hypotheses are likely not mutually exclusive and potentially responsible for the variability observed in this study. Conversely, other reports have described a close agreement, with no significant differences between the RCP and NIRS breakpoints (Murias et al., 2013; Fontana et al., 2015; Keir et al., 2015; Iannetta et al., 2017a), suggesting further investigation is warranted.

Our results, however, do not rule out the concept of disparate tissues reaching a local breakpoint at different times, which cumulatively produce the systemic response. It is possible that the breakpoint at the VL is led by elevated whole-body oxygen during cycling (Boone et al., 2015). As for the deltoid, recruitment during cycling may vary between different riding techniques and riding positions. As such, the presence of a Deoxy-BP at the deltoid may result from (1) an increase in O_2_ extraction to produce work of upper body stabilization with increasing locomotor PO and (2) systemic redistribution of blood flow primarily toward working skeletal muscle, respiratory muscles, the heart, and the brain *via* vasoconstriction of non-priorities tissues (Özyener et al., 2012). Our results highlight the importance of including multiple muscle sites during whole-body exercise testing to investigate the sequential and mechanistic relationships between peripheral and systemic expression of metabolic breakpoints. This relationship is important as it may contribute to field application of wearable NIRS for intensity monitoring for the following reasons: (1) it may not be limited only to primary locomotor muscle measurements and (2) other anatomical locations may be less affected by movement artifacts, providing better signal quality during real-world training and competition. Further investigations should be made to address these points.

For practitioners and athletes, the ability to describe local oxygenation responses at multiple muscle sites may help understand the distribution of muscle recruitment, metabolic supply and demand, and cumulative local fatigue in the context of whole-body exercise. However, from our results, we conclude that using wearable NIRS to determine metabolic breakpoints remains premature, insofar as a high degree of individual variability exist even in well-controlled laboratory conditions. Contextual observations of muscle oxygenation responses and their reliability, apart from the determination of metabolic breakpoints, may still provide relevant information for practitioners to understand an individual athlete’s response to exercise. Such observations could focus on stochastic exercise, recovery and repeated efforts, warm-up, and simulation of sport-specific activities to better understand metabolic demand and individual responses.

## Study Limitations

As mentioned previously, V̇O_2_ was measured with an open-circuit respiratory gas analysis system, with mixed expired gases averaged every 15 s and interpolated to 1 Hz for analysis. Unlike a breath-by-breath system, this could have potentially introduced timing errors into the calculation of MRT that may have contributed to the individual variability observed in this parameter. This in turn could have affected the adjustment of V̇O_2_ and PO at Deoxy-BP and RCP, respectively. For this reason, we chose to report the original time of occurrence for all breakpoints as they were measured during exercise, in addition to calculating the MRT-adjusted V̇O_2_ and PO that were associated with those breakpoints.

The analysis methods chosen for the detection of the NIRS-derived Deoxy-BP in this study and in others are based on well-established methods used to determine ventilatory thresholds (Davis, 1985; Beaver et al., 1986; Murias et al., 2013; Iannetta et al., 2019). The commonly used incremental ramp protocol allows for easy comparison to ventilatory thresholds for identification of intensity domains. However, like all methods of assessing physiological breakpoint, the analysis method is specific to the protocol used. The considerable variability in the expression of individual ventilatory thresholds and deoxygenation breakpoints at locomotor and non-locomotor muscle sites among this well-trained subject group may suggest that different assessment protocols may be useful to further explore the sources of this individual heterogeneity. For example, by design, the relatively fast incremental ramp rate (30 W/min) used in the present study does not allow for the kinetics of either pulmonary ventilation or muscle oxygenation to be fully expressed at any particular workload. It could be that a longer multi-stage “step test” protocol could be used to observe the full expression of oxygenation kinetics across multiple locomotor and non-locomotor muscle sites at progressively increasing workloads, in order to better appreciate the interaction of local metabolic responses and their contribution to systemic measurements like pulmonary gas exchange. Future experiments will be able to optimize exercise assessment protocols to the specific advantages of NIRS, rather than be bound to design constraints imposed by unrelated measurement techniques.

Lastly, the default variable, SmO_2_, generated by the Moxy sensor may be more sensitive to changes in blood flow compared to [HHb+Mb] measured with frequency-domain NIRS (Grassi et al., 2003). Because of this setting, it is possible that the high individual variation seen in our study is related to exercise induced vasodilation (Grassi et al., 2003).

## Conclusion

In conclusion, our findings show that a commercially available wearable NIRS sensor can detect the Deoxy-BP in both VL and deltoid muscles, and that there were no differences between the mean Deoxy-BP and mean RCP; however, the high degree of individual variability suggests caution should be taken when translating between these protocol-dependent metabolic breakpoints. As such, further investigations remain warranted in order for practitioners and athletes to use wearable NIRS to detect physiological breakpoints that may be used to prescribe training, predict race performance pacing, and monitor athlete development, from a single incremental ramp exercise test.

## Data Availability Statement

The raw data supporting the conclusions of this article will be made available by the authors, without undue reservation.

## Ethics Statement

The studies involving human participants were reviewed and approved by UBC Clinical Research Ethics Board. The patients/participants provided their written informed consent to participate in this study.

## Author Contributions

AY and JA have equally contributed to the study design, data acquisition, and write up, under the supervision of MK, and guidance of the remaining listed authors. All authors contributed to the article and approved the submitted version.

## Funding

This study was funded by a Natural Sciences and Engineering Research Council Discovery Grant and an equipment grant from the British Columbia Sport and Exercise Medicine Research Foundation.

## Conflict of Interest

The authors declare that the research was conducted in the absence of any commercial or financial relationships that could be construed as a potential conflict of interest.

## Publisher’s Note

All claims expressed in this article are solely those of the authors and do not necessarily represent those of their affiliated organizations, or those of the publisher, the editors and the reviewers. Any product that may be evaluated in this article, or claim that may be made by its manufacturer, is not guaranteed or endorsed by the publisher.

## References

[ref1] Barstow T. J. (2019). Understanding near Infrared spectroscopy and its application to skeletal muscle research. J. App. Physiol. Am. Physiol. Soc. 126, 1360–1376. doi: 10.1152/japplphysiol.00166.2018 30844336

[ref2] Beaver W. L. Wasserman K. Whipp B. J. (1986). A new method for detecting anaerobic threshold by gas exchange. J. Appl. Physiol. 60, 2020–2027. doi: 10.1152/jappl.1986.60.6.2020, PMID: 3087938

[ref3] Boone J. Barstow T. J. Celie B. Prieur F. Bourgois J. (2014). The impact of pedal rate on muscle oxygenation, muscle activation and whole-body VO_2_ during ramp exercise in healthy subjects. Eur. J. Appl. Physiol. 115, 57–70. doi: 10.1007/s00421-014-2991-x, PMID: 25204279

[ref4] Boone J. Barstow T. J. Celie B. Prieur F. Bourgois J. (2015). The interrelationship between muscle oxygenation, muscle activation, and pulmonary oxygen uptake to incremental ramp exercise: influence of aerobic fitness. Appl. Physiol. Nutr. Metab. 41, 55–62. doi: 10.1139/apnm-2015-0261, PMID: 26701120

[ref5] Caen K. Vermeire K. Bourgois J. G. Boone J. (2018). Exercise thresholds on trial: are they really equivalent? Med. Sci. Sports Exerc. 50, 1277–1284. doi: 10.1249/MSS.0000000000001547, PMID: 29315165

[ref6] Contreras-Briceño F. Espinosa-Ramirez M. Hevia G. Llambias D. Carrasco M. Cerda F.. (2019). Reliability of NIRS portable device for measuring intercostal muscles oxygenation during exercise. J. Sports Sci. 37, 2653–2659. doi: 10.1080/02640414.2019.1653422, PMID: 31419921

[ref7] Crum E. M. O’Connor W. J. Van Loo L. Valckx M. Stannard S. R. (2017). Validity and reliability of the Moxy oxygen monitor during incremental cycling exercise. Eur. J. Sport Sci. 17, 1037–1043. doi: 10.1080/17461391.2017.1330899, PMID: 28557670

[ref8] Davis J. A. (1985). Anaerobic threshold: review of the concept and directions for future research. Med. Sci. Sports Exerc. 17, 6–21. doi: 10.1249/00005768-198502000-00003, PMID: 3884961

[ref9] Feldmann A. Schmitz R. Erlacher D. (2019). Near-infrared spectroscopy-derived muscle oxygen saturation on a 0 to 100% scale: reliability and validity of the Moxy monitor. J. Biomed. Opt. 24, 1–11. doi: 10.1117/1.jbo.24.11.115001, PMID: 31741352 PMC7003144

[ref10] Fontana F. Y. Keir D. A. Bellotti C. De Roia G. F. Murias J. M. Pogliaghi S. (2015). Determination of respiratory point compensation in healthy adults: can non-invasive near-infrared spectroscopy help? J. Sci. Med. Sport 18, 590–595. doi: 10.1016/j.jsams.2014.07.016, PMID: 25153251

[ref11] Grassi B. Pogliaghi S. Rampichini S. Quaresima V. Ferrari M. Marconi C.. (2003). Muscle oxygenation and pulmonary gas exchange kinetics during cycling exercise on-transitions in humans. J. Appl. Physiol. 95, 149–158. doi: 10.1152/japplphysiol.00695.2002, PMID: 12611769

[ref12] Iannetta D. Inglis E. C. Fullerton C. Passfield L. Murias J. M. (2018). Metabolic and performance-related consequences of exercising at and slightly above MLSS. Scand. J. Med. Sci. Sports 28, 2481–2493. doi: 10.1111/sms.13280, PMID: 30120803

[ref13] Iannetta D. Murias J. M. Keir D. A. (2019). A simple method to quantify the V-O 2 mean response time of ramp-incremental exercise. Med. Sci. Sports Exerc. 51, 1080–1086. doi: 10.1249/MSS.0000000000001880, PMID: 30601794

[ref14] Iannetta D. Qahtani A. Maturana F. M. Murias J. M. (2017a). The near-infrared spectroscopy-derived deoxygenated Haemoglobin breaking-point is a repeatable measure That demarcates exercise intensity domains. J. Sci. Med. Sport 20, 873–877. doi: 10.1016/j.jsams.2017.01.237, PMID: 28254143

[ref15] Iannetta D. Qahtani A. Millet G. Y. Murias J. M. (2017b). Quadriceps muscles O_2_ extraction and Emg breakpoints during a ramp incremental test. Front. Physiol. 8:686. doi: 10.3389/fphys.2017.00686, PMID: 28970805 PMC5609583

[ref16] Inglis C. Erin D. I. Murias J. M. (2017). The plateau in the NIRS-derived [HHb] signal near the end of a ramp incremental test does not indicate the upper limit of O_2_ extraction in the Vastus Lateralis. Am. J. Physiol. Regul. Integr. Comp. Physiol. 313, R723–R729. doi: 10.1152/ajpregu.00261.2017, PMID: 28931547 PMC5814694

[ref17] Inglis E. C. Iannetta D. Keir D. A. Murias J. M. (2019). Training-induced changes in the RCP, [HHb]BP and MLSS: evidence of equivalence. Int. J. Sports Physiol. Perform. 15, 119–125. doi: 10.1123/ijspp.2019-0046, PMID: 31034305

[ref18] Keir D. A. Fontana F. Y. Robertson T. C. Murias J. M. Paterson D. H. Kowalchuk J. M.. (2015). Exercise intensity thresholds: identifying the boundaries of sustainable performance. Med. Sci. Sports Exerc. 47, 1932–1940. doi: 10.1249/MSS.0000000000000613 25606817

[ref19] Kirby B. S. Clark D. A. Bradley E. M. Wilkins B. W. (2021). The balance of muscle oxygen supply and demand reveals critical metabolic rate and predicts time to exhaustion. J. Appl. Physiol. 130, 1915–1927. doi: 10.1152/japplphysiol.00058.2021, PMID: 33914662

[ref20] Legrand R. Marles A. Prieur F. Lazzari S. (2007). Related trends in Locomotor and respiratory muscle oxygenation during exercise. Med. Sci. Sports Exerc. 39, 91–100. doi: 10.1249/01.mss.0000241638.90348.67, PMID: 17218889

[ref21] Manchado-Gobatto F. B. Marostegan A. B. Rasteiro F. M. Cirino C. Cruz J. P. Moreno M. A.. (2020). New insights into mechanical, metabolic and muscle oxygenation signals During and After high-intensity tethered running. Sci. Rep. 10, 6336–6314. doi: 10.1038/s41598-020-63297-w, PMID: 32286408 PMC7156678

[ref22] McManus C. J. Collison J. Cooper C. E. (2018). Performance comparison of the MOXY and PortaMon near-infrared spectroscopy muscle Oximeters at rest and during exercise. J. Biomed. Opt. 23, 1–14. doi: 10.1117/1.jbo.23.1.015007, PMID: 29368457

[ref23] Mukaka M. M. (2012). Statistics corner: A guide to appropriate use of correlation coefficient in medical research. J. Med. Assoc. Malawi 24, 69–71. PMID: 23638278 PMC3576830

[ref24] Murias J. M. Keir D. A. Spencer M. D. Paterson D. H. (2013). Sex-related differences in muscle Deoxygenation during ramp incremental exercise. Resp. Physiol. Neurobiol. 189, 530–536. doi: 10.1016/j.resp.2013.08.011, PMID: 23994824

[ref25] Ogata H. Reyihan A. Yano T. (2004). Kinetics of oxygenation in inactive forearm muscle during ramp leg cycling. J. Physiol. Anthropol. Appl. Hum. Sci. 23, 7–17. doi: 10.2114/jpa.23.7, PMID: 14757996

[ref26] Özyener F. Whipp B. J. Ward S. A. (2012). The contribution of ‘resting’ body muscles to the slow component of pulmonary oxygen uptake during high-intensity cycling. J. Sports Sci. Med. 11, 759–767. PMID: 24150089 PMC3763325

[ref27] Perrey S. Ferrari M. (2018). Muscle oximetry in sports science: a systematic review. Sports Med. 48, 597–616. doi: 10.1007/s40279-017-0820-1 29177977

[ref28] Racinais S. Buchheit M. Girard O. Perrey S. (2014). Breakpoints in ventilation, cerebral and muscle oxygenation, and muscle activity during an incremental cycling exercise. Front. Physiol. 5:142. doi: 10.3389/fphys.2014.00142, PMID: 24782786 PMC3990045

[ref29] Rodrigo-Carranza V. González-Mohíno F. Turner A. P. Rodriguez-Barbero S. González-Ravé J. M. (2021). Using a portable near-infrared spectroscopy device to estimate the second Ventilatory threshold. Int. J. Sports Med. 42, 905–910. doi: 10.1055/a-1343-2127, PMID: 33525000

[ref30] Shiroishi K. Kime R. Osada T. Murase N. Shimomura K. Katsumura T. (2010). Decreased muscle oxygenation and increased arterial blood flow in the non-exercising limb during leg exercise. Adv. Exp. Med. Biol. 662, 379–384. doi: 10.1007/978-1-4419-1241-1_55, PMID: 20204819

[ref32] Tanaka H. Shimizu S. Ohmori F. Muraoka Y. Kumagai M. Yoshizawa M.. (2006). Increases in blood flow and shear stress to nonworking limbs during incremental exercise. Med. Sci. Sports Exerc. 38, 81–85. doi: 10.1249/01.mss.0000191166.81789.de, PMID: 16394957

[ref33] Zwaard S. Jaspers R. T. Blokland I. J. Achterberg C. Visser J. M. Den Uil A. R.. (2016). Oxygenation threshold derived from near- infrared spectroscopy: reliability and its relationship with the first Ventilatory threshold. PLoS One 11, 1–16. doi: 10.1371/journal.pone.0162914, PMID: 27631607 PMC5025121

